# Reliability and Validity of Lumbar-Mounted IMU for Postural Sway Assessment in Healthy Adults

**DOI:** 10.3390/s26216677

**Published:** 2026-10-22

**Authors:** Giuseppe Prisco, Noemi Pisani, Francesco Amato, Fabrizio Esposito, Leandro Donisi

**Affiliations:** 1 Department of Advanced Medical and Surgical Sciences, University of Campania Luigi Vanvitelli, 80138 Naples, Italy; giuseppe.prisco95@gmail.com (G.P.); noemi.pisani@unicampania.it (N.P.); fabrizio.esposito@unicampania.it (F.E.); 2 Department of Information Technology and Electrical Engineering, University of Naples Federico II, 80125 Naples, Italy; framato@unina.it

**Keywords:** postural control, quiet standing, stabilometry, wearable sensors

## Abstract

Inertial measurement units (IMUs) are increasingly used as portable tools for postural sway assessment, for which force platforms are the gold standard. This study aimed to evaluate the short-term test–retest reliability, concurrent validity, and construct validity of postural sway metrics derived from a lumbar-mounted IMU, using centre of pressure (CoP) measures obtained from a force platform as the reference standard. Sixty-nine healthy adults performed two quiet standing conditions, with eyes open (EO) and eyes closed (EC), while lumbar centre of mass (CoM) acceleration and CoP displacement signals were recorded simultaneously. Ten postural sway metrics in both the time and frequency domains were extracted from the CoM and CoP signals. The IMU-based approach demonstrated moderate-to-good reliability for most time-domain parameters. Mean velocity exhibited the highest reliability (intraclass correlation coefficient, ICC = 0.779 in EO and 0.813 in EC), comparable to that obtained with the force platform. Moderate-to-strong correlations were found between IMU- and force platform-derived measures for distance-, velocity-, and area-related metrics (r = 0.52 to 0.73). Furthermore, both approaches detected significant differences in postural sway between the EO and EC conditions, supporting their sensitivity to changes in sensory input. Frequency-domain parameters showed lower reliability and weaker associations, and systematic differences between the two approaches were found for several parameters. These findings suggest that lumbar-mounted IMUs may provide a practical approach for assessing specific postural sway metrics, particularly mean velocity and distance-related parameters, when force platform assessment is not feasible, although the two approaches should not be considered interchangeable.

## 1. Introduction

Postural control is a fundamental motor function in maintaining balance and adapting to both internal and external perturbations [1,2]. Even during quiet standing, balance is maintained through continuous postural adjustment mechanisms, which arise from the integration of visual, vestibular, and somatosensory inputs, coupled with appropriate neuromuscular responses [3]. These subtle body oscillations, commonly referred to as postural sway, are indicative of the functional integrity of the postural control system, and their assessment is widely employed to evaluate balance performance in both healthy individuals and clinical populations [4]. Stabilometry, defined as the quantitative analysis of postural sway, constitutes a well-established methodology for the assessment of postural control. Specifically, the analysis of the centre of pressure (CoP) trajectories during quiet standing provides detailed information on sway amplitude, spatial dispersion, velocity, and spectral content, all of which are considered indicators of balance performance and postural stability [5,6].

To date, force platforms are considered the gold standard for posturographic assessments owing to their accuracy and high temporal resolution and are widely used in biomechanical research and clinical practice [7,8,9]. However, force platforms are subject to inherent practical limitations, notably their lack of portability and limited adaptability to real-world assessment settings, which restrict their applicability to controlled laboratory environments [10].

In recent years, wearable inertial measurement units (IMUs), which integrate accelerometers, gyroscopes, and magnetometers, have emerged as a promising tool for postural sway assessment, particularly in contexts requiring evaluations outside laboratory settings. Their use is especially relevant in telemedicine, where remote and home-based monitoring of motor function has gained increasing clinical importance [11,12,13,14,15,16].

However, a key distinction between the two measurement modalities, namely force platforms and inertial sensors, lies in the physical nature of the signals they acquire. Unlike force platforms, which directly measure the CoP as the point of application of the ground reaction forces, IMUs capture inertial signals that primarily reflect the kinematics of the body’s centre of mass (CoM), particularly when positioned on the lumbar region. Although CoM and CoP are functionally related, they characterise the postural control system at different levels of analysis: the CoM reflects the overall outcome of body movements, whereas the CoP is more closely associated with the neuromuscular control actions involved in regulating and correcting those movements [17,18]. Under quasi-static conditions, the CoP trajectory oscillates continuously around, and often beyond, the projection of the CoM onto the support surface in order to generate the horizontal ground reaction forces required to decelerate and stabilise CoM displacement. Consequently, CoP and CoM are indirectly related and reflect the behaviour of the postural control system under specific biomechanical assumptions [17,19].

Given these differences, it remains unclear to what extent postural sway metrics derived from CoM-related inertial signals can accurately reproduce the measurements typically obtained from CoP data, particularly considering that the two signals belong to different physical domains, namely acceleration and displacement. Furthermore, methodological factors, such as signal processing techniques and biomechanical modelling approaches, may further influence the computation of sway parameters [20,21]. Therefore, the validity and reliability of IMU-derived postural sway metrics require systematic evaluation before their implementation in clinical and research settings.

Several studies have evaluated the feasibility of postural sway metrics derived from IMUs during quiet standing tasks, using force platform measures as the gold standard for comparison [18,22,23,24,25]. Mayagoitia et al. [18] investigated the construct validity across four experimental conditions in a sample of 8 healthy male participants, extracting five time-domain and frequency-domain metrics from a lumbar-mounted IMU. Their results showed that accelerometer-derived metrics were able to discriminate between balance conditions, with performance comparable to that of force platform-based measures. Whitney et al. [22] examined the reliability and concurrent validity of three time-domain sway parameters in a larger cohort of 81 healthy subjects assessed under the Sensory Organization Test (SOT) protocol. Statistically significant correlations were observed between pelvis accelerometry and CoP measures, suggesting that accelerometer-derived metrics provide balance-related information comparable to that obtained from traditional CoP measures. Similarly, Mancini et al. [23] evaluated the reliability and concurrent validity of 13 time-domain and frequency-domain parameters in 17 healthy subjects assessed under the Instrumented Sway (ISway) test, further corroborating the strong associations between lumbar IMU-based and CoP-derived sway metrics. However, the substantial heterogeneity across studies, in terms of sample size, methodological approach, and the selection of extracted metrics, limits the generalisability of the findings. Consequently, the validity of IMU-based posturography cannot yet be considered fully established.

In this context, the present study aimed to determine whether a lumbar-mounted IMU can provide reliable and valid measures of postural sway comparable to those obtained from a force platform. Specifically, short-term test–retest reliability, concurrent validity, and construct validity were evaluated for a set of time-domain and frequency-domain sway metrics derived from IMU-based CoM-related signals, with CoP measures obtained from a force platform serving as the reference standard. Assessments were conducted under two quiet standing conditions: eyes open (EO) and eyes closed (EC). To ensure methodological consistency between the two measurement approaches (IMU vs force platform), the same metric model was applied to both types of signals, enabling the extraction of equivalent time-domain and frequency-domain parameters from CoM-related acceleration signals and CoP displacement signals.

## 2. Materials and Methods

### 2.1. IMU-Based System

**Figure 1:**
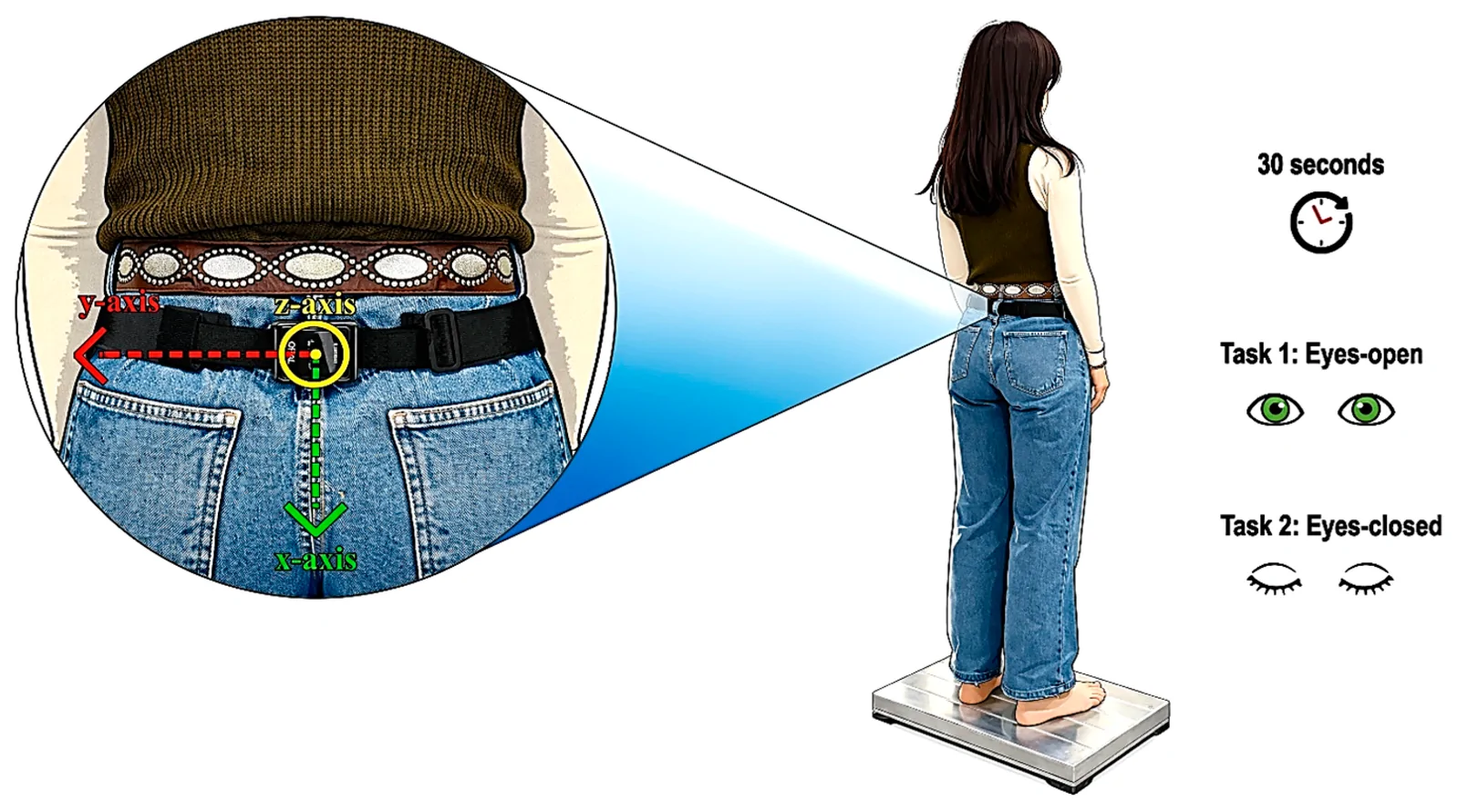
Experimental setup: force platform; IMU and local coordinate frames; test timing; test conditions (eyes-open and eyes-closed).

The Mobility Lab system (APDM Inc., Portland, OR, USA) is a commercially available wearable system based on IMUs for movement and posturographic analyses. The system comprises IMUs (Opal sensors), an access point, a docking station, and proprietary software for data acquisition and analysis (Moveo Explorer). The IMUs acquire linear acceleration and angular velocity signals at a sampling frequency of 128 Hz. Sensor data are synchronously transmitted to a host computer via the access point using the Bluetooth 3.0 communication protocol, whereas the docking station is used for sensor charging and configuration. The Moveo Explorer software (Ver 1.0.0.20226161718) supports data acquisition, processing, and storage according to standardised assessment protocols. In the present study, a single IMU was positioned on the lumbar region, in proximity to the body CoM (Figure 1).

### 2.2. Force Platform System

The BTS P-6000 (BTS Bioengineering, Milan, Italy) is a triaxial force platform designed for stabilometric analysis and quantitative assessment of postural control (Figure 1). The platform features an active area of 600 × 400 mm, a height of 50 mm, and a total mass of 28 kg. It incorporates strain-gauge force transducers with a 16-bit resolution and a measurement range of ±2000 N along each orthogonal axis. The system enables the acquisition of the three orthogonal components of the ground reaction force vector and the CoP displacements at a sampling frequency of 1000 Hz. Data acquisition, visualisation, and storage are managed by proprietary software (BTS Analyzer, Ver 1.10), which allows real-time monitoring and recording of force and CoP signals.

### 2.3. Study Population

Sample size was verified for the two main analyses: for test–retest reliability, using the Intraclass Correlation Coefficient (ICC) method [26] with a target ICC of 0.80, a null-hypothesis ICC of 0.60, two measurements per participant, a two-sided significance level of 0.05, and 90% statistical power (β = 0.10), a minimum of 66 participants was required. For concurrent validity, using Spearman correlation analysis [27] with a target correlation of 0.40, a two-sided significance level of 0.05, and 90% statistical power (β = 0.10), a minimum of 67 participants was required. Accordingly, sixty-nine healthy volunteers (23 males and 46 females) were enrolled in this study. Participants’ age, height, and body mass are reported as mean ± standard deviation (SD): 26.21 ± 4.22 years, 168.65 ± 8.78 cm, and 67.87 ± 13.71 kg, respectively. Inclusion criteria were age ≥18 years and the ability to stand independently without assistive devices. Exclusion criteria comprised a history of balance disorders, orthopaedic or neurological conditions affecting postural control, and current use of medications known to influence postural stability. The experimental protocol was conducted in accordance with the Declaration of Helsinki, and written informed consent was obtained from all participants.

### 2.4. Experimental Setup and Study Protocol

A single IMU was placed at the level of the fifth lumbar vertebra (L5), as a proxy for the body’s CoM, and secured with the manufacturer’s elastic strap. Before data collection, participants were familiarised with the experimental setup and tasks. During all trials, participants stood barefoot on the force platform with arms relaxed alongside the body and head facing forward. Foot placement followed the Feet Apart condition of the Mobility Lab ISway protocol. As prescribed by the protocol, the feet were aligned using the manufacturer’s plastic foot alignment plate (medial heel-to-heel distance of 10 cm), which was removed before recording. Two 30 s quiet standing tasks were performed: eyes open (EO), with participants fixating a black cross at eye level on the wall 1.5 m in front of the platform, and eyes closed (EC). Participants were instructed to stand as still as possible and not to speak during recording. Each participant completed two acquisition sessions on the same day, separated by 5 min of rest. Within each session, the EO and EC tasks were performed consecutively in a fixed order (EO followed by EC) for all participants. During the rest interval, the IMU was removed, recalibrated, reconfigured, and repositioned to minimise sensor drift. All trials were supervised by the same evaluator, who delivered identical instructions across sessions. The experimental setup and stabilometric tasks are illustrated in Figure 1.

### 2.5. Signal Processing and Postural Sway Parameter Extraction

Only the anteroposterior (AP) and mediolateral (ML) components of the IMU linear acceleration and of the CoP displacement were processed, corresponding to the z- and y-axes, respectively, of the reference frames shown in Figure 1. The two systems were synchronised manually: the operator started the force platform recording upon an audio cue automatically emitted by the Mobility Lab system at the onset of each IMU recording.

**Figure 2:**
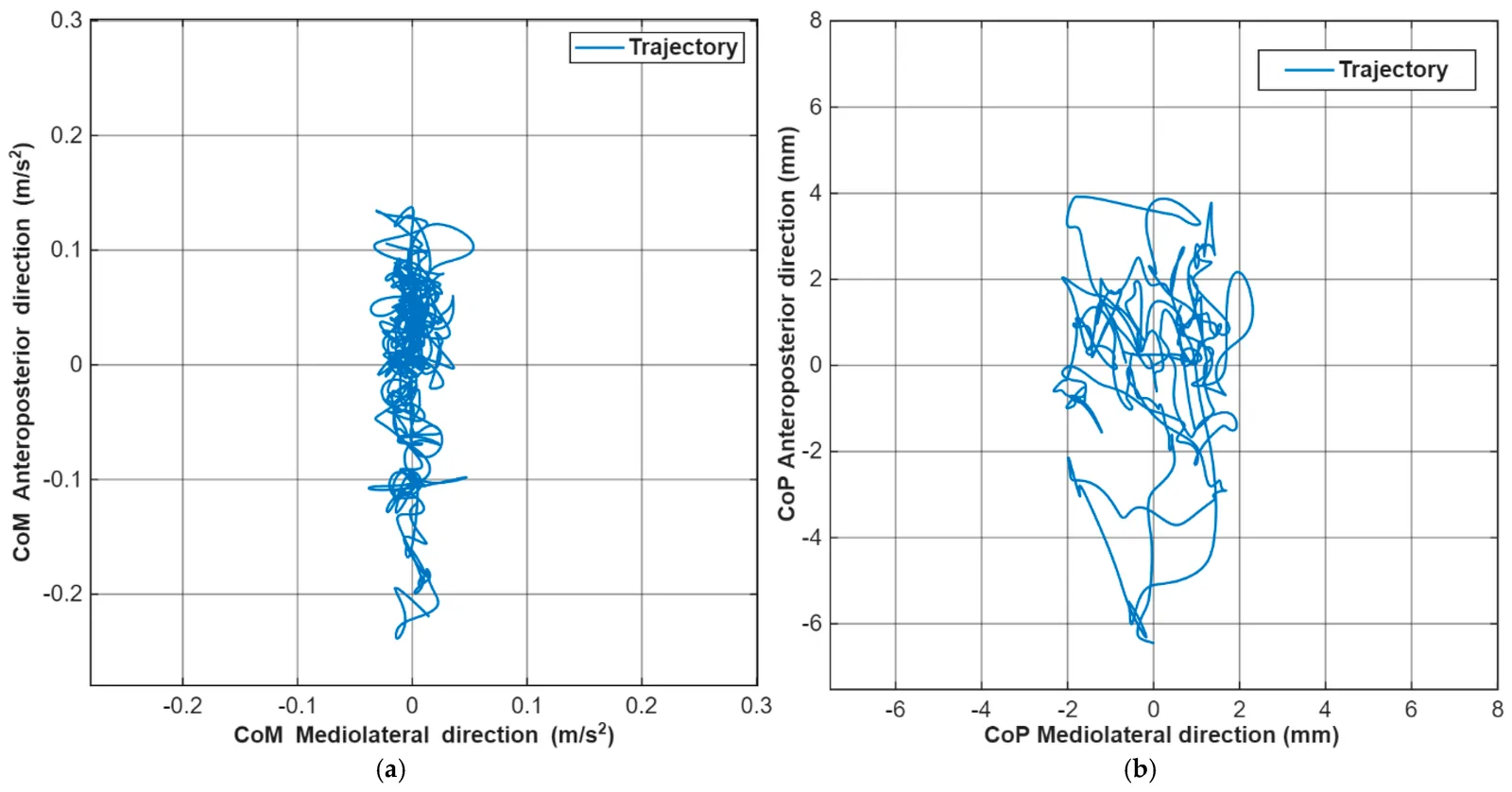
Representative stabilograms obtained during the eyes-open condition. (**a**) Stabilogram in the acceleration domain derived from CoM anteroposterior and mediolateral lumbar acceleration measured by the lumbar-mounted IMU. (**b**) Stabilogram in the displacement domain derived from anteroposterior and mediolateral CoP displacement measured by the force platform.

CoP signals were down-sampled from 1000 Hz to 128 Hz to match the IMU sampling frequency, using the MATLAB 2025a (MathWorks, Natick, MA, USA) resample function, which applies a polyphase anti-aliasing FIR filter. To minimise edge artefacts, each CoP series was padded at both ends with time-reversed segments before resampling, and the padding was removed afterwards. This procedure preserves the frequency content of postural sway, which lies predominantly below 3 Hz [28]. Both signals were then low-pass filtered with a fourth-order Butterworth filter (cutoff frequency 5 Hz) to attenuate high-frequency noise. For IMU acceleration signals, the mean value of each AP and ML time series was subtracted before parameter extraction, thus removing the constant gravitational component associated with the static inclination of the sensor. The filtered AP and ML signals were then used to obtain an acceleration-domain stabilogram for the IMU (Figure 2a) and a displacement-domain stabilogram for the force platform (Figure 2b).

For both stabilograms, a set of postural sway parameters was extracted in the time-domain and frequency-domain. Time-domain metrics were computed as follows:

- Mean distance (MDIST):

(1) MDIST=1N∑n=1NRD(n)

where the resultant distance RD(n) is defined as:

(2) $$RD\left(n\right)={\left[{AP(n)}^{2}+{ML(n)}^{2}\right]}^{1/2}$$

- Range of distance (RDIST):

(3) $$RDIST=\left|\max\left(RD\right)-min(RD)\right|$$

- Root mean square of distance (RMS-DIST):

(4) RMSDIST=1N∑n=1NRD(n)21/2

- Mean velocity (MV):

(5) MV=TOTEXT

where the total excursion TOTEX is given by:

(6) $$TOTEX=\sum_{n=1}^{N-1}{\left[{\left(AP\left(n+1\right)-AP(n)\right)}^{2}+{\left(ML\left(n+1\right)-ML(n)\right)}^{2}\right]}^{1/2}$$

- Sway area (SA):

(7) $$SA=\frac{1}{2T}\sum_{n=1}^{N-1}\left|AP\left(n+1\right)ML\left(n\right)-AP(n)ML(n+1)\right|$$

- Ellipse area at the 95% confidence level (CEA):

(8) $$CEA=\pi ab=2\pi F_{0.05[2,n-2]}{\left[s_{AP}^{2}s_{ML}^{2}-s_{AP\;ML}^{2}\right]}^{1/2}$$

- Ellipse eccentricity (ε):

(9) $$\varepsilon =\sqrt{\frac{a^{2}-b^{2}}{a^{2}}}$$

where the semi-major and semi-minor axes of the 95% confidence ellipse are defined as:

(10) $$a={\left[F_{0.05\left[2,n-2\right]}(s_{AP}^{2}+s_{ML}^{2}+D)\right]}^{1/2}\;\;\;b={\left[F_{0.05\left[2,n-2\right]}(s_{AP}^{2}+s_{ML}^{2}-D)\right]}^{1/2}$$

and D is the discriminant of the covariance matrix:

(11) $$D={\left[(s_{AP}^{2}+s_{ML}^{2})-4(s_{AP}^{2}s_{ML}^{2}-s_{AP\;ML}^{2})\right]}^{1/2}$$

Frequency-domain parameters were derived from the power spectral density (PSD), denoted as G(m), of both CoM acceleration and CoP displacement signals. The PSD was estimated using the fast Fourier transform (FFT) algorithm. The following frequency-domain metrics were computed:

- Mean frequency (MF):

(12) $$MF=\frac{TOTEX}{2\pi MDIST\cdot T}$$

- Centroidal frequency (CF):

(13) CF=μ2μ01/2

where $\mu_{k}$ is

(14) $$\mu_{k}=\sum_{m=1}^{N}{\left(m∆f\right)}^{k}G(m)$$

- Dispersion frequency (DF):

(15) DF=1−μ12μ0μ21/2

where

- AP(n) is the n-th sample of the AP time series;
- ML(n) is the n-th sample of the ML time series;
- N is the number of samples of the signal;
- RD is the resultant distance;
- TOTEX is the total length of the path;
- $F_{0.05\left[2,n-2\right]}$ is the F statistic at a 95% confidence level for a bivariate distribution with n data points;
- $s_{AP}$ is the SD of the AP time series;
- $s_{ML}$ is the SD of the ML time series;
- $s_{AP\;ML}$ is the covariance between the AP and ML time series;
- a is the major radius of the 95% confidence level ellipse;
- b is the minor radius of the 95% confidence level ellipse;
- D is the discriminant of the covariance matrix;
- T is the duration of the task;
- m is the m-th sample of the signal in the frequency domain;
- G(m) is the discrete power spectral density of RD;
- Δf is the frequency increment of G(m);
- μ_k_ is the k-th spectral moment.

In all equations, AP(n) and ML(n) correspond to either CoM linear acceleration signals acquired from the IMU or CoP displacement signals acquired from the force platform, depending on the stabilogram under analysis.

Although the original formulations of these parameters were developed for CoP-based stabilograms [29], the present study extended their application to acceleration signals acquired from a lumbar-mounted IMU as an indirect measure of CoM dynamics. This approach is supported by previous studies reporting significant associations between postural sway metrics derived from lumbar-mounted inertial sensors and corresponding CoP-based measures [30,31].

### 2.6. Statistical Analysis

The statistical analysis addressed three objectives: (i) the short-term test–retest reliability of the parameters obtained with each system, (ii) the concurrent validity of the IMU-derived parameters against the force platform, and (iii) the construct validity of each system across sensory conditions (EO vs. EC). As the Shapiro–Wilk test indicated non-normal distributions for all parameters, non-parametric methods were used throughout.

Short-term test–retest reliability was quantified with the ICC(3,1), computed with a two-way mixed-effects model for the consistency of single measurements, together with its 95% confidence interval (CI) [32]. ICC values were interpreted as poor (<0.50), moderate (0.50 to 0.75), good (0.75 to 0.90), or excellent (>0.90). Absolute reliability was quantified with the normalised standard error of measurement (SEM) and the normalised minimal detectable change (MDC) at the 95% confidence level, both reported as a percentage of the grand mean (SEM% and MDC%) [33,34]. Systematic differences between sessions were examined using Bland–Altman (BA) analysis.

Concurrent validity was evaluated in terms of the association between IMU- and force platform-derived sway parameters. The association between the two approaches was quantified for each parameter using Spearman’s rank correlation coefficient (r) [33], interpreting the r values as weak (0.10 to 0.39), moderate (0.40 to 0.69), strong (0.70 to 0.89), or very strong (≥0.90). Since the two systems yield parameters in different physical domains, systematic differences for each sway parameter were assessed on the EC/EO ratio. EC/EO ratios were compared between the two methods using the Wilcoxon signed-rank test. Finally, Passing–Bablok (PB) regression was applied to the EC/EO ratios to detect constant and proportional systematic errors, indicating the presence of these two errors if the 95% CI of the intercept does not include zero and the 95% CI of the slope does not include 1, respectively [34].

Construct validity was assessed as the ability of each system to detect the expected increase in postural sway when vision is removed. For each parameter and system, EO and EC values were compared with the Wilcoxon signed-rank test, and the effect size was quantified with the matched-pairs rank-biserial correlation (r_β_) [35].

Statistical significance was set at α = 0.05. Benjamini–Hochberg false discovery rate correction for multiple comparisons was applied separately within each family of analyses (concurrent validity, EC/EO ratio comparisons, construct validity). All analyses were performed in MATLAB R2025b (MathWorks, Natick, MA, USA).

## 3. Results

### 3.1. Short-Term Test–Retest Reliability

Table 1 and Table 2 report the short-term test–retest reliability of the postural sway parameters for both systems in the EO and EC conditions, respectively. Tables S1 and S2 also report the 95% CI of the BA bias.

### 3.2. Concurrent Validity

**Figure 3:**
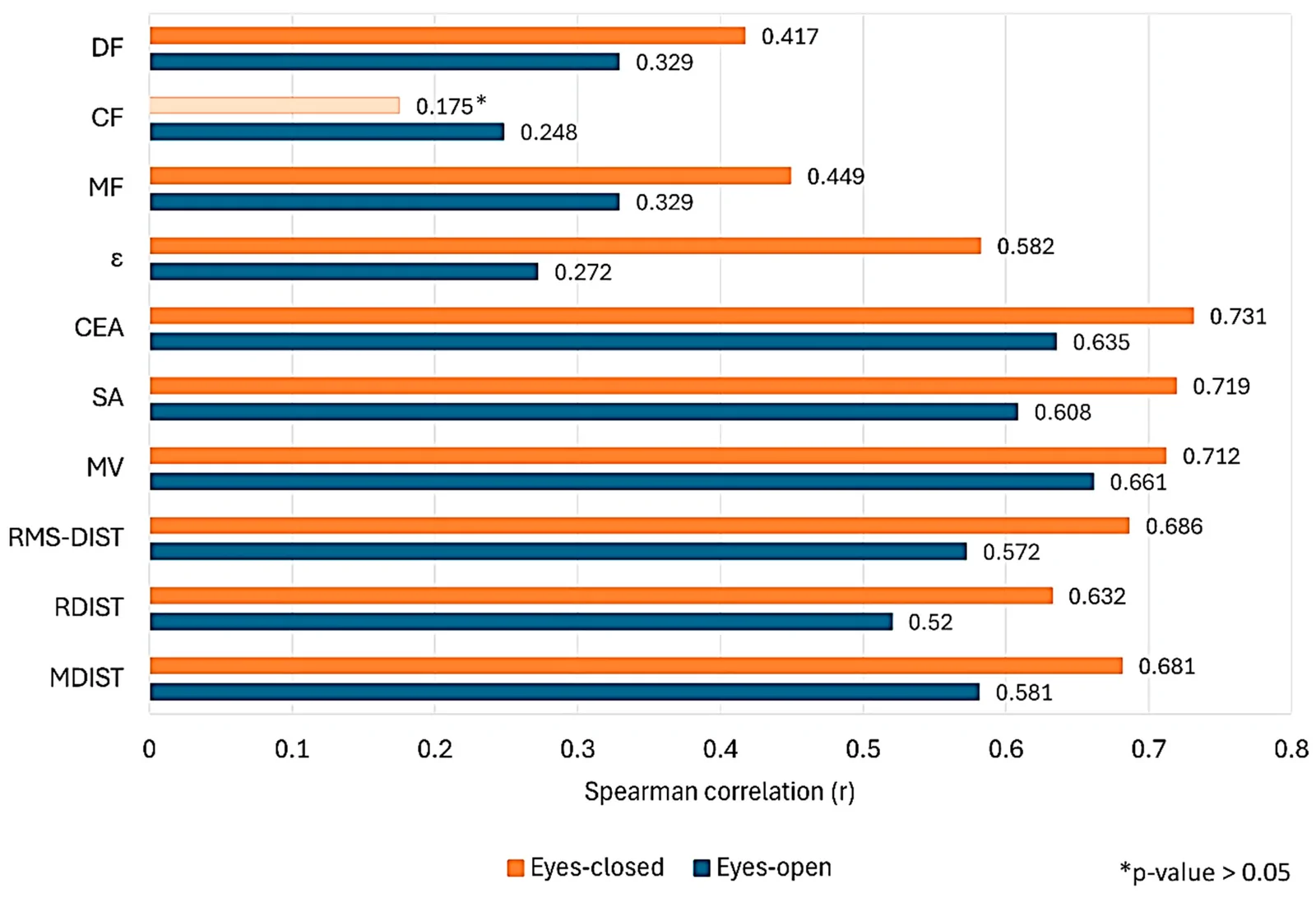
Spearman correlation coefficients (r) between IMU- and force platform-derived postural sway parameters in the eyes-open (blue) and eyes-closed (orange) conditions. Transparent bars indicate non-significant correlations (* adjusted *p*-value > 0.05).

Figure 3 shows the Spearman correlation coefficients between systems for each parameter in the EO and EC conditions, and Tables S3 and S4 report the corresponding descriptive statistics and adjusted *p*-values. Table 3 compares the EC/EO ratios between systems, and Table 4 reports the PB regression of the EC/EO ratios.

### 3.3. Construct Validity

**Figure 4:**
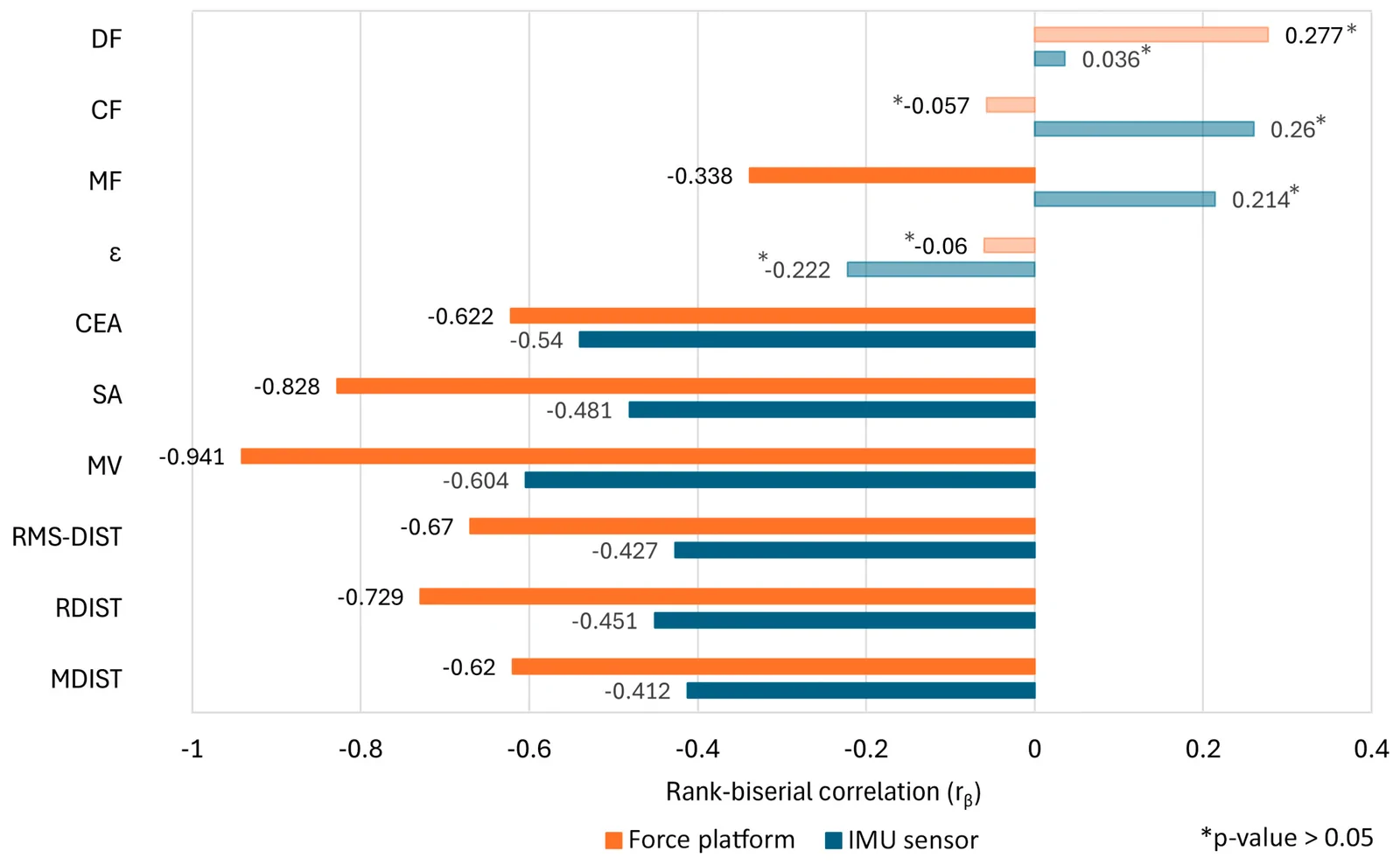
Effect size of the eyes-open versus eyes-closed comparison, expressed as the matched-pairs rank-biserial correlation (r_β_), for postural sway parameters derived from the IMU (blue) and the force platform (orange). Transparent bars indicate non-significant differences (* adjusted *p*-value > 0.05).

Figure 4 shows, for each parameter and system, the effect size (r_β_) and the significance of the EO versus EC comparison. Tables S5 and S6 report the descriptive statistics, Wilcoxon signed-rank test results, and r_β_ for the IMU and the force platform, respectively.

## 4. Discussion

The present study investigated the ability of a single lumbar-mounted IMU to assess postural sway during quiet standing. Specifically, three complementary methodological objectives were addressed: (i) evaluating short-term test–retest reliability of each postural sway parameter, (ii) assessing concurrent validity against a force platform as the reference standard, and (iii) examining construct validity across different sensory conditions, namely EO and EC.

### 4.1. Short-Term Test–Retest Reliability

The reliability analysis showed that, for most postural sway parameters, the lumbar-mounted IMU achieved reliability comparable to that of the force platform.

For the IMU, MV showed the highest ICC in both conditions: 0.779 (95% CI: 0.667 to 0.856) in EO (Table 1) and 0.813 (95% CI: 0.716 to 0.880) in EC (Table 2). The distance-related metrics MDIST and RMS-DIST showed moderate ICC values in EO (0.510 and 0.526, respectively; Table 1), which increased to 0.674 and 0.655 in EC (Table 2). Area-based parameters, which typically show greater variability [29], also improved in EC: the ICC increased from 0.421 to 0.642 for SA and from 0.465 to 0.604 for CEA (Table 1 and Table 2). These findings broadly agree with previous studies that investigated the reliability of lumbar-mounted IMUs during quiet standing in both EO and EC conditions. For instance, Mancini et al. [23] used the ISway protocol to evaluate the reliability of time-domain and frequency-domain parameters derived from a lumbar-mounted IMU in healthy individuals. Time-domain parameters yielded ICC values ranging from 0.70 to 0.89, whereas frequency-domain parameters showed lower reliability (0.25 < ICC < 0.60). The same pattern was observed in the present study, except for MF, which showed moderate-to-good reliability (ICC = 0.624 in EO and 0.763 in EC). Similarly, Hansson et al. [36] reported moderate-to-high reliability (0.60 < ICC < 0.85) for MV in AP and ML directions across repeated sessions in healthy adults, in agreement with the present findings. Compared with the studies by Mancini et al. [23] and Hansson et al. [36], a methodological strength of the present study is its larger sample size, which yields more precise reliability estimates. Moe-Nilssen et al. [24] reported ICC values between 0.40 and 0.55 for RMS-DIST in the AP direction during quiet standing, with reliability generally higher in EC than EO conditions, consistent with the present results. In the present study, the EC reliability of RMS-DIST (ICC = 0.655) exceeded that reported by Moe-Nilssen et al. [24]. Finally, the poor-to-moderate ICC values for area-based parameters (SA and CEA) are in line with Heebner et al. [25], who reported greater between-session variability for area-based metrics than distance- and velocity-based metrics, even in healthy populations.

For the force platform approach, MV was also the most reliable parameter, with ICC values of 0.692 in EO (Table 1) and 0.729 in EC (Table 2), slightly lower than the corresponding IMU values. Distance-based parameters, such as RDIST, showed poor-to-moderate reliability, with ICC values of 0.505 in EO and 0.440 in EC (Table 1 and Table 2, respectively). Area-based parameters, such as SA, showed moderate reliability, with ICC values of 0.548 in EO and 0.594 in EC (Table 1 and Table 2, respectively). By contrast, frequency-domain metrics differed between the two approaches: whereas the IMU yielded poor-to-good reliability (ICC ranging from 0.357 for DF in EO to 0.763 for MF in EC), force platform-derived CF and DF showed poor reliability in both conditions (ICC ≤ 0.183), with 95% CIs including zero. Similarly poor reliability was observed for force platform-derived ε in EO (ICC = 0.035). Frequency-domain parameters are generally considered more sensitive than spatial parameters to noise and methodological differences, as previously reported by Prieto et al. [29].

In addition, absolute reliability was assessed with SEM% and MDC% at the 95% confidence level, which, being expressed relative to the grand mean, allows comparison of the relative measurement variability of the two approaches despite their different physical units. For the IMU approach, MV showed the lowest measurement error among the distance-, velocity-, and area-based parameters in both conditions, with SEM% values of 16.19% in EO and 14.79% in EC, and MDC% of 44.87% and 41.00%, respectively (Table 1 and Table 2). Distance-related parameters exhibited lower SEM% and MDC% values in EC than in EO: for MDIST, SEM% and MDC% values decreased from 25.44% and 70.51% in EO (Table 1) to 20.09% and 55.67% in EC (Table 2), and for RMS-DIST from 25.42% and 70.46% to 20.77% and 57.56%, respectively. Area-based parameters showed the largest relative measurement errors but followed a similar trend: for SA, SEM% and MDC% decreased from 62.05% and 172.00% in EO to 33.81% and 93.71% in EC, and for CEA from 64.15% and 177.82% to 35.77% and 99.16%, respectively (Table 1 and Table 2). These findings are consistent with those reported by Mancini et al. [23] and Heebner et al. [25] for balance assessment using inertial systems. For the force platform approach, MV also showed the lowest measurement error, with an SEM% of 14.13% and an MDC% of 39.18% in EO (Table 1), which increased slightly to 15.95% and 44.21% in EC (Table 2); these values were comparable to those obtained with the IMU. Distance-based parameters showed SEM% values between 19.65% and 23.98% and MDC% values between 54.47% and 66.46% in both conditions, with only minor changes between EO and EC (Table 1 and Table 2). The largest measurement errors were observed for area-based parameters, particularly for CEA (SEM% = 42.14% and MDC% = 116.80% in EO; 47.46% and 131.56% in EC), reflecting the high variability of CEA estimates and their sensitivity to CoP excursions, a limitation previously reported in posturographic studies [8,29]. In the EO, area-based parameters showed larger relative measurement errors with the IMU than with the force platform, whereas in EC the SEM% and MDC% values of the two systems were comparable. The very low SEM% and MDC% values observed for DF (≤0.21% and ≤0.58%, respectively) and, to a lesser extent, for ε should not be interpreted as an indication of high measurement precision. Rather, they mainly reflect the small variability of these bounded parameters relative to their mean value, as also suggested by their poor-to-moderate ICC values.

The BA analysis showed that the 95% CI of the bias included zero for most parameters and conditions, indicating the absence of statistically significant constant systematic errors between the two sessions. Regarding the IMU approach in the EO condition (Table 1 and Table S1), the 95% CI of the bias included zero for all parameters, indicating the absence of constant systematic error, and the LOA were centred close to zero (e.g., MDIST: −4.260 × 10^−2^ to 4.260 × 10^−2^ m/s^2^; MV: −0.139 to 0.154 m/s). However, the LOA corresponded approximately to the MDC% values, indicating that between-session differences at the individual level were substantial, in line with the moderate-to-good ICC values. These relative values were comparable to those of the force platform, suggesting that this variability is largely intrinsic to short quiet-standing recordings rather than specific to the IMU. The same pattern was observed in EC (Table 2 and Table S2), where all biases were non-significant, and the LOA were generally lower than in EO, although still substantial. For the force platform approach in EO (Table 1 and Table S1), three parameters showed statistically significant biases, as their 95% CIs did not include zero: MDIST (0.367 mm; 95% CI: 0.044 to 0.691), RMS-DIST (0.380 mm; 95% CI: 0.026 to 0.733), and SA (1.007 mm^2^; 95% CI: 0.037 to 1.977), indicating a minimal but consistent constant systematic error between sessions. In EC (Table 2 and Table S2) all biases were not statistically significant, with 95% CIs uniformly encompassing zero. CEA showed wide LOA in both conditions (EO: −135.424 to 171.523 mm^2^; EC: −266.594 to 247.021 mm^2^), reflecting high between-session variability in ellipse-area estimation.

Overall, the ICC values obtained for the IMU approach were comparable to those obtained using the force platform approach across most postural sway parameters, suggesting that IMU-derived metrics may achieve a level of test–retest consistency comparable to that of the gold standard force platform, despite being derived from a different measurement domain.

### 4.2. Concurrent Validity

The concurrent validity analysis showed moderate-to-strong correlations between the IMU and the force platform approaches for most postural sway parameters in both EO and EC conditions.

All distance-, velocity-, and area-based parameters were significantly correlated between systems in both conditions (adjusted *p*-value < 0.001; Tables S3 and S4), whereas ε showed only a weak correlation in EO (r = 0.272) and a moderate correlation in EC (r = 0.582; Figure 3). Among these parameters, MV showed the strongest correlations in EO (r = 0.661) and one of the strongest in EC (r = 0.712; Figure 3), indicating that velocity-based parameters are among the most consistently aligned across measurement domains. Distance-based parameters showed moderate correlations in both conditions: r = 0.581 in EO and r = 0.681 in EC for MDIST, 0.572 and 0.686 for RMS-DIST, and 0.520 and 0.632 for RDIST (Figure 3). Area-based metrics showed moderate-to-strong correlations: r = 0.608 in EO and r = 0.719 in EC for SA, r = 0.635 in EO and r = 0.731 in EC for CEA (Figure 3). Overall, correlations for time-domain parameters consistently increased from EO to EC, likely because the removal of visual input increases the magnitude of postural oscillations, making the association between acceleration- and displacement-based metrics more detectable. In contrast, frequency-domain parameters showed weak-to-moderate correlations (Tables S3 and S4). In particular, r was 0.329 in EO (adjusted *p*-value = 0.007) and 0.449 in EC (adjusted *p*-value < 0.001) for MF, and 0.329 (adjusted *p*-value = 0.008) and 0.417 (adjusted *p*-value < 0.001) for DF; CF showed the lowest values, with r = 0.248 in EO (adjusted *p*-value = 0.039) and a non-significant r = 0.175 in EC (adjusted *p*-value = 0.147). These findings are broadly consistent with previous validation studies comparing postural sway parameters derived from lumbar-mounted IMU and force platforms. In healthy adults assessed with the SOT, Whitney et al. [22] reported weak-to-moderate correlations for RMS-DIST (r = 0.15 in EO; r = 0.45 in EC) and RDIST (r = 0.21 in EO; r = 0.57 in EC), substantially lower than those obtained in the present study. This discrepancy might be attributable to differences in the acquisition protocol, as the SOT includes both static and dynamic tasks under visual perturbations, whereas the present study employed only static tasks without sensory perturbations. Whitney et al. [22] also reported stronger correlations in EC than in EO, a pattern reproduced in the present study for all time-domain parameters. Similarly, Hansson et al. [36] reported strong correlations for MV (r = 0.71 in EO; r = 0.81 in EC) in healthy subjects during 30 s of quiet standing, further supporting the consistency of the present findings.

Additionally, the EC/EO ratios were compared between the two systems using the Wilcoxon signed-rank test to determine whether the two approaches differed in their sensitivity to visual deprivation, independently of the overall magnitude of postural sway. The EC/EO ratios of most parameters differed significantly between the two systems (adjusted *p*-value < 0.05; Table 3), with the force platform consistently exhibiting higher ratios than the IMU. This difference was most pronounced for MV (force platform: 1.424 ± 0.332 vs. IMU: 1.122 ± 0.200; adjusted *p*-value < 0.001), SA (1.842 ± 0.947 vs. 1.396 ± 0.659; adjusted *p*-value < 0.001), and CEA (1.857 ± 1.124 vs. 1.493 ± 0.768; adjusted *p*-value < 0.001), reflecting the greater sensitivity of displacement-based parameters to the biomechanical consequences of visual deprivation. Significant differences were also observed for MDIST (1.332 ± 0.480 vs. 1.236 ± 0.442; adjusted *p*-value = 0.022), RMS-DIST (1.341 ± 0.476 vs. 1.249 ± 0.457; adjusted *p*-value = 0.018), MF (1.171 ± 0.394 vs. 1.001 ± 0.327; adjusted *p*-value < 0.001), and CF (1.231 ± 0.743 vs. 0.990 ± 0.435; adjusted *p*-value = 0.024; Table 3), suggesting that spectral parameters also partially reflect the different sensitivity of the two systems to visual deprivation. Overall, these findings indicate that force platform-derived metrics were more sensitive to visual deprivation than lumbar acceleration-based metrics, possibly because the slow, large-amplitude postural oscillations that characterize EC standing produce large increases in CoP displacement but smaller increases in CoM acceleration. The EC/EO ratios of ε, RDIST, and DF did not differ significantly between the two systems (adjusted *p*-value = 0.594, 0.216, and 0.239, respectively; Table 3), indicating no detectable difference in the relative postural response for these parameters.

PB regression analysis further supported these observations (Table 4). For most parameters, the 95% CI of the slope did not include 1, indicating a proportional systematic error whereby the IMU-based EC/EO ratios increased less than those of the force platform. This was most evident for MV (m = 0.487, 95% CI: 0.347 to 0.644), SA (m = 0.580, 95% CI: 0.450 to 0.787), CEA (m = 0.600, 95% CI: 0.499 to 0.729), CF (m = 0.368, 95% CI: 0.210 to 0.598), ε (m = 0.407, 95% CI: 0.298 to 0.609), and DF (m = 0.528, 95% CI: 0.360 to 0.814), whose 95% CIs were all entirely below 1. Conversely, for MDIST (m = 0.832, 95% CI: 0.658 to 1.039), RMS-DIST (m = 0.867, 95% CI: 0.700 to 1.106), and MF (m = 0.752, 95% CI: 0.551 to 1.020), the slope was lower than 1 but its 95% CI included 1, indicating no statistically significant proportional difference between systems (Table 4). This proportional difference may be related to the different measurement domains of the two systems: while displacement-based CoP metrics capture the overall spatial excursion of postural sway, acceleration-based metrics mainly reflect the dynamic component of CoM motion and may therefore attenuate some of the low-frequency, large-amplitude adjustments that are more evident in EC. RDIST was the only parameter with a slope exceeding 1 (m = 1.164, 95% CI: 0.796 to 1.717), even though its 95% CI included 1, indicating that no statistically significant proportional difference was found for this parameter either. The 95% CI of the intercept included 0 for MDIST, RDIST, RMS-DIST, SA, and MF, suggesting the absence of significant constant bias for these parameters. Conversely, the intercept differed significantly from 0 for MV (q = 0.442, 95% CI: 0.203 to 0.605), CEA (q = 0.314, 95% CI: 0.118 to 0.455), ε (q = 0.591, 95% CI: 0.395 to 0.702), CF (q = 0.525, 95% CI: 0.277 to 0.690), and DF (q = 0.472, 95% CI: 0.186 to 0.640), pointing to a combination of proportional and constant systematic error for these metrics (Table 4).

Overall, the IMU and the force platform approaches showed moderate-to-strong associations for time-domain postural sway parameters. However, the EC/EO analysis revealed systematic differences between systems that also involved time-domain parameters such as MV, SA, and CEA, and PB regression showed neither proportional nor constant bias only for MDIST, RDIST, RMS-DIST, and MF.

### 4.3. Construct Validity

The construct validity analysis showed significant differences in postural sway parameters between EO and EC conditions for both the IMU and the force platform approaches. Regarding the IMU approach, the Wilcoxon test revealed statistically significant differences in MDIST, RDIST, RMS-DIST, MV, SA, and CEA when visual input was removed (adjusted *p*-value ≤ 0.003; Figure 4, Table S5). These differences were accompanied by moderate to large effect sizes, with r_β_ ranging from −0.412 (MDIST) to −0.604 (MV), indicating a consistent increase in postural sway in EC (Figure 4). Frequency-domain parameters and ε did not show statistically significant differences, suggesting limited sensitivity of these metrics to changes in sensory conditions (Figure 4, Table S5). Similarly, the force platform approach showed significant increases in all distance-, velocity-, and area-based parameters between EO and EC conditions (adjusted *p*-value < 0.001), with larger effect sizes (r_β_: −0.620 to −0.941; Figure 4, Table S6). Conversely, ε and the frequency-domain parameters showed no statistically significant changes, except for MF, which increased slightly but significantly (adjusted *p*-value = 0.014, r_β_ = −0.338). CF and DF remained stable across conditions (adjusted *p*-value > 0.05; Figure 4, Table S6). These findings are consistent with the sensorimotor integration framework described by Peterka [4], according to which the removal of visual feedback prompts the postural control system to reweight its reliance toward vestibular and somatosensory inputs, a compensatory mechanism that, under quiet standing conditions, is associated with increased postural oscillations across both measurement domains. This pattern also reflects principles of sensory organization theory: during quiet standing, the central nervous system continuously integrates visual, vestibular, and somatosensory inputs to update its internal model of the CoM trajectory [37,38]. Vision provides a high-resolution negative feedback signal that attenuates postural oscillations; its removal therefore results in larger, less constrained sway. The present results are further corroborated by the normative dataset of Prieto et al. [29], who reported significant increases in distance-, velocity-, and area-based CoP parameters with eye closure, with MV showing the largest and most consistent effect. In the present study, MV likewise showed the largest effect size for both the IMU approach (r_β_ = −0.604; Figure 4) and the force platform approach (r_β_ = −0.941; Figure 4). However, the magnitude of the EO–EC effect differed substantially between the two systems. The force platform consistently yielded larger effect sizes across time-domain parameters (r_β_: −0.620 to −0.941) compared to the IMU method (r_β_: −0.412 to −0.604). This attenuation of the IMU response likely reflects a fundamental difference in what the two systems measure. Force platforms quantify CoP displacement, which directly captures the spatial excursion associated with increased postural sway under visual deprivation. In contrast, IMUs measure lumbar acceleration, which reflects the rate of change in CoM motion rather than its absolute displacement. Consequently, the large and slow postural oscillations typically observed in EC may produce substantial increases in displacement but only modest increases in acceleration. This difference systematically attenuates the relative magnitude of the EO–EC changes measured by the IMU compared with the force platform. Finally, the absence of significant EO–EC differences for frequency-domain parameters in the present study was in line with evidence from Prieto et al. [29] and Ghislieri et al. [20], who noted that spectral metrics derived from IMUs were less sensitive to sensory condition manipulations than time-domain measures, particularly in young healthy populations where postural frequency content remains largely stable across conditions.

The ability of the IMU system to detect these expected physiological responses supports its construct validity, as it reflects sensitivity to expected changes in sensory conditions. The pronounced changes in time-domain parameters were consistent with the physiological understanding that removal of visual input destabilises postural control, while minimal alterations in frequency-domain metrics suggested these parameters are less influenced by sensory condition changes during quiet standing.

## 5. Conclusions

This study investigated whether a single lumbar-mounted IMU could serve as a reliable and valid measurement approach comparable to the force platform, considered the gold standard in stabilometric analysis, for the assessment of postural sway during quiet standing in healthy adults.

Regarding short-term test–retest reliability, the IMU approach demonstrated moderate-to-good reliability for most sway parameters across both EO and EC conditions, with test–retest reliability comparable to that of the force platform. MV exhibited the highest reliability in both systems, while frequency-domain parameters generally showed lower and less stable reliability, particularly when derived from the force platform, except for MF.

With respect to concurrent validity, moderate-to-strong correlations were observed between the two approaches for time-domain parameters, with correlation strength generally increasing under EC conditions. In contrast, frequency-domain parameters showed weak-to-moderate correlations, with CF showing no significant correlation in the EC condition. However, systematic differences between systems were found for several parameters, including MV, SA, and CEA, reflecting the different physical quantities captured by each system.

Concerning construct validity, both methods successfully demonstrated sensitivity to changes in sensory conditions from EO to EC. Moderate-to-large effect sizes were observed for the IMU approach, whereas larger effect sizes were found for the force platform approach. Frequency-domain parameters did not show significant EO–EC differences for the IMU.

Overall, these findings suggest that lumbar-mounted IMUs provide a practical approach for assessing specific postural sway metrics when force platform assessment is not feasible, although the two approaches measure different physical quantities and their outputs should not be considered interchangeable. MV showed the highest test–retest reliability and one of the strongest associations with force platform measurements; however, the presence of both proportional and constant bias indicates that its values cannot be directly comparable between systems. MDIST and RMS-DIST also showed moderate-to-strong associations with force platform measurements, without statistically detectable systematic bias in the EC/EO ratios. However, agreement and interchangeability between the two systems remain unestablished. In contrast, area-based parameters and most frequency-domain parameters should be interpreted with caution, given their weaker associations and/or poorer agreement with force platform measurements.

The present study is not without limitations. First, it included only young healthy individuals, thereby limiting the generalisability of the findings to older adults and clinical populations, in whom balance impairments are more prevalent and clinically relevant. Second, the experimental protocol was restricted to short-duration (30 s), static quiet standing tasks under EO and EC conditions, which may not fully capture the complexity of postural control in real-world or clinical scenarios. Third, both sessions were performed on the same day with a short rest interval and a fixed EO–EC order.

Future investigations should extend the validation of IMU-based sway metrics to older adults and individuals with neurological or musculoskeletal conditions to better clarify their clinical applicability. Furthermore, the inclusion of dynamic balance tasks, dual-task paradigms, perturbed stance conditions, and longer recording durations may help elucidate differences between acceleration-based and displacement-based systems that were not addressed in the present study.

## Figures and Tables

**Table 1:** Short-term test–retest reliability of postural sway parameters derived from the IMU and the force platform in the eyes-open condition: intraclass correlation coefficient (ICC) with 95% confidence interval (CI), standard error of measurement (SEM%) and minimal detectable change at the 95% confidence level (MDC%) expressed as percentages of the grand mean, and Bland–Altman bias and limits of agreement (LOA).

**Short-term test–retest reliability IMU sensor**					
				**Bland–Altman analysis**	
**Postural sway** **parameters**	**ICC (95%CI)**	**SEM%**	**MDC%**	**bias**	**LOA**
MDIST	0.510 (0.313 to 0.665)	25.436	70.506	−2.714 × 10^−5^	−4.260 × 10^−2^ to 4.260 × 10^−2^
RDIST	0.536 (0.345 to 0.684)	23.901	66.250	3.400 × 10^−3^	−0.163 to 0.169
RMS-DIST	0.526 (0.332 to 0.677)	25.420	70.462	4.971 × 10^−4^	−4.900 × 10^−2^ to 5.000 × 10^−2^
MV	0.779 (0.667 to 0.856)	16.187	44.868	7.300 × 10^−3^	−0.139 to 0.154
SA	0.421 (0.211 to 0.595)	62.053	172.003	1.100 × 10^−3^	−1.110 × 10^−2^ to 1.330 × 10^−2^
CEA	0.465 (0.261 to 0.630)	64.153	177.824	3.100 × 10^−3^	−4.410 × 10^−2^ to 5.040 × 10^−2^
ε	0.292 (0.062 to 0.493)	6.783	18.803	6.000 × 10^−3^	−0.206 to 0.218
MF	0.624 (0.456 to 0.749)	22.009	61.006	1.100 × 10^−2^	−0.684 to 0.706
CF	0.541 (0.351 to 0.688)	23.177	64.244	8.760 × 10^−2^	−2.415 to 2.590
DF	0.357 (0.133 to 0.545)	0.071	0.196	−7.857 × 10^−5^	−2.500 × 10^−3^ to 2.300 × 10^−3^
**Short-term test–retest reliability force platform**					
				**Bland–Altman analysis**	
**Postural sway** **parameters**	**ICC (95%CI)**	**SEM%**	**MDC%**	**bias**	**LOA**
MDIST	0.459 (0.256 to 0.625)	23.975	66.455	0.367	−2.339 to 3.074
RDIST	0.505 (0.310 to 0.660)	19.650	54.466	0.801	−7.009 to 8.611
RMS-DIST	0.454 (0.250 to 0.620)	22.867	63.383	0.380	−2.577 to 3.336
MV	0.692 (0.547 to 0.797)	14.133	39.175	0.137	−2.733 to 3.007
SA	0.548 (0.362 to 0.692)	36.005	99.801	1.007	−7.107 to 9.121
CEA	0.546 (0.360 to 0.691)	42.137	116.798	18.049	−135.424 to 171.523
ε	0.035 (−0.204 to 0.269)	8.657	23.997	5.300 × 10^−3^	−0.285 to 0.296
MF	0.526 (0.336 to 0.676)	21.232	58.853	−2.510 × 10^−2^	−0.245 to 0.195
CF	0.034 (−0.202 to 0.266)	26.977	74.777	0.120	−2.097 to 2.338
DF	0.036 (−0.200 to 0.268)	0.161	0.445	3.400 × 10^−4^	−5.800 × 10^−3^ to 6.500 × 10^−3^

Abbreviations: CEA = ellipse area at the 95% confidence level; CF = centroidal frequency; CI = confidence interval; DF = dispersion frequency; ICC = intraclass correlation coefficient; LOA = limits of agreement; MDC% = minimal detectable change at the 95% confidence level as a percentage; MDIST = mean distance; MF = mean frequency; MV = mean velocity; RDIST = range of distance; RMS-DIST = root mean square of distance; SA = sway area; SEM% = standard error of measurement as a percentage; ε = ellipse eccentricity.

**Table 2:** Short-term test–retest reliability of postural sway parameters derived from the IMU and the force platform in the eyes-closed condition: intraclass correlation coefficient (ICC) with 95% confidence interval (CI), standard error of measurement (SEM%) and minimal detectable change at the 95% confidence level (MDC%) expressed as percentages of the grand mean, and Bland–Altman bias and limits of agreement (LOA).

**Short-term test–retest reliability IMU sensor**					
				**Bland–Altman analysis**	
**Postural sway** **parameters**	**ICC (95%CI)**	**SEM%**	**MDC%**	**bias**	**LOA**
MDIST	0.674 (0.522 to 0.784)	20.085	55.673	8.857 × 10^−5^	−3.700 × 10^−2^ to 3.720 × 10^−2^
RDIST	0.421 (0.207 to 0.596)	21.930	60.787	−4.400 × 10^−3^	−0.192 to 0.183
RMS-DIST	0.655 (0.497 to 0.771)	20.765	57.557	−1.657 × 10^−4^	−4.570 × 10^−2^ to 4.530 × 10^−2^
MV	0.813 (0.716 to 0.880)	14.793	41.004	2.500 × 10^−3^	−0.144 to 0.149
SA	0.642 (0.480 to 0.762)	33.807	93.707	−4.094 × 10^−5^	−7.300 × 10^−3^ to 7.300 × 10^−3^
CEA	0.604 (0.430 to 0.734)	35.773	99.159	−7.100 × 10^−4^	−3.240 × 10^−2^ to 3.090 × 10^−2^
ε	0.475 (0.272 to 0.637)	3.868	10.721	−7.100 × 10^−3^	−0.123 to 0.108
MF	0.763 (0.645 to 0.846)	16.502	45.742	2.320 × 10^−2^	−0.445 to 0.491
CF	0.455 (0.248 to 0.623)	23.990	66.497	8.020 × 10^−2^	−2.282 to 2.443
DF	0.540 (0.353 to 0.686)	0.209	0.580	−5.143 × 10^−4^	−7.100 × 10^−3^ to 6.000 × 10^−3^
**Short-term test–retest reliability force platform**					
				**Bland–Altman analysis**	
**Postural sway** **parameters**	**ICC (95%CI)**	**SEM%**	**MDC%**	**bias**	**LOA**
MDIST	0.447 (0.238 to 0.617)	22.652	62.789	3.810 × 10^−2^	−3.090 to 3.166
RDIST	0.440 (0.233 to 0.610)	21.268	58.952	0.906	−10.222 to 12.033
RMS-DIST	0.471 (0.266 to 0.635)	21.651	60.014	8.900 × 10^−2^	−3.362 to 3.540
MV	0.729 (0.598 to 0.823)	15.948	44.206	0.218	−4.343 to 4.780
SA	0.594 (0.417 to 0.727)	35.579	98.620	−0.282	−13.034 to 12.470
CEA	0.527 (0.334 to 0.677)	47.463	131.560	−9.786	−266.594 to 247.021
ε	0.346 (0.123 to 0.536)	6.782	18.799	1.260 × 10^−2^	−0.190 to 0.215
MF	0.623 (0.456 to 0.748)	20.221	56.049	8.100 × 10^−3^	−0.233 to 0.250
CF	0.067 (−0.173 to 0.298)	32.515	90.126	−4.100 × 10^−2^	−2.638 to 2.556
DF	0.183 (−0.046 to 0.395)	0.132	0.366	−5.100 × 10^−4^	−5.200 × 10^−3^ to 4.200 × 10^−3^

Abbreviations: CEA = ellipse area at the 95% confidence level; CF = centroidal frequency; CI = confidence interval; DF = dispersion frequency; ICC = intraclass correlation coefficient; LOA = limits of agreement; MDC% = minimal detectable change at the 95% confidence level as a percentage; MDIST = mean distance; MF = mean frequency; MV = mean velocity; RDIST = range of distance; RMS-DIST = root mean square of distance; SA = sway area; SEM% = standard error of measurement as a percentage; ε = ellipse eccentricity.

**Table 3:** Eyes-closed to eyes-open (EC/EO) ratios of postural sway parameters derived from the IMU and the force platform: mean ± standard deviation (SD) for each system and adjusted *p*-value of the Wilcoxon signed-rank test between systems.

Postural Sway Parameters	IMU Sensor Mean ± SD	Force Platform Mean ± SD	Adjusted *p*-Value
MDIST	1.236 ± 0.442	1.332 ± 0.480	0.022
RDIST	1.286 ± 0.519	1.344 ± 0.459	0.216
RMS-DIST	1.249 ± 0.457	1.341 ± 0.476	0.018
MV	1.122 ± 0.200	1.424 ± 0.332	<0.001
SA	1.396 ± 0.659	1.842 ± 0.947	<0.001
CEA	1.493 ± 0.768	1.857 ± 1.124	<0.001
ε	1.046 ± 0.145	1.028 ± 0.199	0.594
MF	1.001 ± 0.327	1.171 ± 0.394	<0.001
CF	0.990 ± 0.435	1.231 ± 0.743	0.024
DF	1.000 ± 0.002	1.000 ± 0.003	0.239

Abbreviations: CEA = ellipse area at the 95% confidence level; CF = centroidal frequency; DF = dispersion frequency; MDIST = mean distance; MF = mean frequency; MV = mean velocity; RDIST = range of distance; RMS-DIST = root mean square of distance; SA = sway area; SD = standard deviation; ε = ellipse eccentricity.

**Table 4:** Passing–Bablok regression of the eyes-closed to eyes-open (EC/EO) ratios of postural sway parameters, with the IMU as the dependent variable and the force platform as the independent variable: slope (m) and intercept (q) with their 95% confidence intervals (CI).

Postural Sway Parameters	m	95% CIm	q	95% CIq
MDIST	0.832	0.658 to 1.039	0.099	−0.161 to 0.285
RDIST	1.164	0.796 to 1.717	−0.246	−0.956 to 0.196
RMS-DIST	0.867	0.700 to 1.106	0.040	−0.214 to 0.252
MV	0.487	0.347 to 0.644	0.442	0.203 to 0.605
SA	0.580	0.450 to 0.787	0.299	−0.071 to 0.464
CEA	0.600	0.499 to 0.729	0.314	0.118 to 0.455
ε	0.407	0.298 to 0.609	0.591	0.395 to 0.702
MF	0.752	0.551 to 1.020	0.143	−0.129 to 0.342
CF	0.368	0.210 to 0.598	0.525	0.277 to 0.690
DF	0.528	0.360 to 0.814	0.472	0.186 to 0.640

Abbreviations: 95% CIm = 95% confidence interval of m; 95% CIq = 95% confidence interval of q; CEA = ellipse area at the 95% confidence level; CF = centroidal frequency; DF = dispersion frequency; MDIST = mean distance; MF = mean frequency; MV = mean velocity; RDIST = range of distance; RMS-DIST = root mean square of distance; SA = sway area; ε = ellipse eccentricity.

## Data Availability

The datasets generated and analyzed in this study are not publicly available due to the privacy policy but are available from the corresponding author upon reasonable request.

## Supplementary Materials

The following supporting information can be downloaded at: https://www.mdpi.com/article/10.3390/s26216677/s1; Table S1: Bland–Altman analysis for the test–retest reliability of postural sway parameters derived using the IMU sensor and force platform during the eyes-open task, reporting bias, 95% confidence interval (CI), and limits of agreement (LOA); Table S2: Bland–Altman analysis for the test–retest reliability of postural sway parameters derived using the IMU sensor and the force platform during the eyes-closed task, reporting bias, 95% confidence interval (CI), and limits of agreement (LOA); Table S3: Concurrent validity of postural sway parameters derived using the IMU sensor and the force platform during the eyes-open condition, and reporting mean ± standard deviation (SD) values for each system, together with the Spearman correlation coefficient (r) and corresponding adjusted *p*-value; Table S4: Concurrent validity of postural sway parameters derived using the IMU sensor and the force platform during the eyes-closed condition, and reporting the mean ± standard deviation (SD) values for each system, together with the Spearman correlation coefficient (r) and corresponding adjusted *p*-value; Table S5: Construct validity of postural sway parameters derived using the IMU sensor during eyes-open and eyes-closed conditions, reporting mean ± standard deviation (SD) values together with results of the Wilcoxon signed-rank test (adjusted *p*-value) and rank-biserial correlation coefficients (r_β_); Table S6: Construct validity of postural sway parameters derived using the force platform during eyes-open and eyes-closed conditions, reporting mean ± standard deviation (SD) values together with results of the Wilcoxon signed-rank test (adjusted *p*-value) and rank-biserial correlation coefficients (r_β_).

## Abbreviations

The following abbreviations are used in this manuscript:

AP	Anteroposterior
BA	Bland–Altman
CEA	Ellipse area
CF	Centroidal frequency
CI	Confidence interval
CoM	Centre of mass
CoP	Centre of pressure
DF	Dispersion frequency
EC	Eyes-closed
EO	Eyes-open
FFT	Fast Fourier transform
ICC	Intraclass correlation coefficient
IMUs	Inertial measurement units
LOA	Limits of agreement
MDC	Minimal detectable change
MDIST	Mean distance
MF	Mean frequency
ML	Mediolateral
m	Slope of the Passing–Bablok linear regression line
MV	Mean velocity
PB	Passing–Bablok
PSD	Power spectral density
q	Intercept of the Passing–Bablok linear regression line
r	Spearman correlation coefficient
rβ	Rank-biserial correlation coefficients
RD	Resultant distance
RDIST	Range of distance
RMS-DIST	Root mean square of distance
SA	Sway area
SD	Standard deviation
SEM	Standard error of measurement
SOT	Sensory organization test
TOTEX	Total excursion
ε	Ellipse eccentricity
