# Supplementary material for: Reliability and Validity of Lumbar-Mounted IMU for Postural Sway Assessment in Healthy Adults
Source: Sensors (Basel). 2026 Oct 22;26(21):6677. doi: 10.3390/s26216677 (PMC13765922; doi:10.3390/s26216677)
Supplement: Supplementary file 1 [file sensors-26-06677-s001.zip › sensors-4576265-supplementary.pdf]

Table S1. Bland–Altman analysis for the test–retest reliability of postural sway parameters derived using the IMU sensor and force platform during the eyes-open task, reporting bias, 95% confidence interval (CI), and limits of agreement (LOA).

**Test–retest reliability IMU sensor**			
**Postural sway parameters**	**Bias**	**95% CI Bias**	**LOA**
MDIST	-2.714×10^{-5}	-5.100×10^{-3} to 5.100×10^{-3}	-4.260×10^{-2} to 4.260×10^{-2}
RDIST	3.400×10^{-3}	-1.640×10^{-2} to 2.320×10^{-2}	-0.163 to 0.169
RMS-DIST	4.971×10^{-4}	-5.400×10^{-3} to 6.400×10^{-3}	-4.900×10^{-2} to 5.000×10^{-2}
MV	7.300×10^{-3}	-1.020×10^{-2} to 2.480×10^{-2}	-0.139 to 0.154
SA	1.100×10^{-3}	-3.450×10^{-4} to 2.600×10^{-3}	-1.110×10^{-2} to 1.330×10^{-2}
CEA	3.100×10^{-3}	-2.500×10^{-3} to 8.800×10^{-3}	-4.410×10^{-2} to 5.040×10^{-2}
ε	6.000×10^{-3}	-1.930×10^{-2} to 3.140×10^{-2}	-0.206 to 0.218
MF	1.100×10^{-2}	-7.210×10^{-2} to 9.400×10^{-2}	-0.684 to 0.706
CF	8.760×10^{-2}	-0.212 to 0.387	-2.415 to 2.590
DF	-7.857×10^{-5}	-3.620×10^{-4} to 2.050×10^{-4}	-2.500×10^{-3} to 2.300×10^{-3}
**Test–retest reliability force platform**			
**Postural sway parameters**	**Bias**	**95% CI Bias**	**LOA**
MDIST	0.367	4.390×10^{-2} to 0.691	-2.339 to 3.074
RDIST	0.801	-0.132 to 1.734	-7.009 to 8.611
RMS-DIST	0.380	2.620×10^{-2} to 0.733	-2.577 to 3.336
MV	0.137	-0.206 to 0.480	-2.733 to 3.007
SA	1.007	3.690×10^{-2} to 1.977	-7.107 to 9.121
CEA	18.049	-0.294 to 36.393	-135.424 to 171.523
ε	5.300×10^{-3}	-2.940×10^{-2} to 4.000×10^{-2}	-0.285 to 0.296
MF	-2.510×10^{-2}	-5.140×10^{-2} to 1.200×10^{-3}	-0.245 to 0.195
CF	0.120	-0.144 to 0.385	-2.097 to 2.338
DF	3.400×10^{-4}	-3.980×10^{-4} to 1.100×10^{-3}	-5.80×10^{-3} to 6.500×10^{-3}

Abbreviations: CEA = ellipse area at the 95% confidence level; CF = centroidal frequency; DF = dispersion frequency; LOA = limits of agreement; MDIST = mean distance; MF = mean frequency; MV = mean velocity; RDIST = range of distance; RMS-DIST = root mean square of distance; SA = sway area; ε = ellipse eccentricity.

Table S2. Bland–Altman analysis for the test–retest reliability of postural sway parameters derived using the IMU sensor and the force platform during the eyes-closed task, reporting bias, 95% confidence interval (CI), and limits of agreement (LOA).

Postural sway parameters	Bias	95% CI Bias	LOA
**Test–retest reliability IMU sensor**			
MDIST	8.857×10^{-5}	-4.300×10^{-3} to 4.500×10^{-3}	-3.700×10^{-2} to 3.720×10^{-2}
RDIST	-4.400×10^{-3}	-2.680×10^{-2} to 1.800×10^{-2}	-0.192 to 0.183
RMS-DIST	-1.657×10^{-4}	-5.600×10^{-3} to 5.300×10^{-3}	-4.570×10^{-2} to 4.530×10^{-2}
MV	2.500×10^{-3}	-1.510×10^{-2} to 2.000×10^{-2}	-0.144 to 0.149
SA	-4.094×10^{-5}	-9.140×10^{-4} to 8.320×10^{-4}	-7.300×10^{-3} to 7.300×10^{-3}
CEA	-7.100×10^{-4}	-4.500×10^{-3} to 3.100×10^{-3}	-3.240×10^{-2} to 3.090×10^{-2}
ε	-7.100×10^{-3}	-2.100×10^{-2} to 6.700×10^{-3}	-0.123 to 0.108
MF	2.320×10^{-2}	-3.280×10^{-2} to 7.910×10^{-2}	-0.445 to 0.491
CF	8.020×10^{-2}	-0.202 to 0.363	-2.282 to 2.443
DF	-5.143×10^{-4}	-1.300×10^{-3} to 2.690×10^{-4}	-7.100×10^{-3} to 6.000×10^{-3}
**Test–retest reliability force platform**			
**Postural sway parameters**	**Bias**	**95% CI Bias**	**LOA**
MDIST	3.810×10^{-2}	-0.336 to 0.412	-3.090 to 3.166
RDIST	0.906	-0.424 to 2.236	-10.222 to 12.033
RMS-DIST	8.900×10^{-2}	-0.323 to 0.501	-3.362 to 3.540
MV	0.218	-0.327 to 0.763	-4.343 to 4.780
SA	-0.282	-1.806 to 1.242	-13.034 to 12.470
CEA	-9.786	-40.481 to 20.908	-266.594 to 247.021
ε	1.260×10^{-2}	-1.160×10^{-2} to 3.670×10^{-2}	-0.190 to 0.215
MF	8.100×10^{-3}	-2.070×10^{-2} to 3.700×10^{-2}	-0.233 to 0.250
CF	-4.100×10^{-2}	-0.351 to 0.269	-2.638 to 2.556
DF	-5.100×10^{-4}	-1.100×10^{-3} to 5.000×10^{-5}	-5.200×10^{-3} to 4.200×10^{-3}

Abbreviations: CEA = ellipse area at the 95% confidence level; CF = centroidal frequency; DF = dispersion frequency; LOA = limits of agreement; MDIST = mean distance; MF = mean frequency; MV = mean velocity; RDIST = range of distance; RMS-DIST = root mean square of distance; SA = sway area; ε = ellipse eccentricity.

Table S3. Concurrent validity of postural sway parameters derived using the IMU sensor and the force platform during the eyes-open condition, reporting mean ± standard deviation (SD) values for each system, together with the Spearman correlation coefficient (r) and corresponding adjusted p-value.

Postural sway parameters	IMU sensor Mean ± SD	Force platform Mean ± SD	Spearman correlation	
			r	Adjusted p-value
MDIST	0.052 ± 0.021	3.357 ± 1.132	0.581	< 0.001
RDIST	0.217 ± 0.077	12.146 ± 3.569	0.520	< 0.001
RMS-DIST	0.061 ± 0.023	3.848 ± 1.270	0.572	< 0.001
MV	0.303 ± 0.099	6.654 ± 1.718	0.661	< 0.001
SA	0.006 ± 0.003	6.762 ± 3.542	0.608	< 0.001
CEA	0.021 ± 0.013	108.075 ± 71.935	0.635	< 0.001
ε	0.900 ± 0.098	0.868 ± 0.103	0.272	0.026
MF	1.016 ± 0.412	0.343 ± 0.125	0.329	0.007
CF	3.364 ± 1.323	2.072 ± 0.774	0.248	0.039
DF	0.997 ± 0.001	0.997 ± 0.003	0.329	0.008

Abbreviations: CEA = ellipse area at the 95% confidence level; CF = centroidal frequency; DF = dispersion frequency; MDIST = mean distance; MF = mean frequency; MV = mean velocity; r = Spearman correlation coefficient; RDIST = range of distance; RMS-DIST = root mean square of distance; SA = sway area; SD = standard deviation; ε = ellipse eccentricity.

Table S4. Concurrent validity of postural sway parameters derived using the IMU sensor and the force platform during the eyes-closed condition, reporting the mean ± standard deviation (SD) values for each system, together with the Spearman correlation coefficient (r) and corresponding adjusted p-value.

Postural sway parameters	IMU sensor Mean ± SD	Force platform Mean ± SD	Spearman correlation	
			r	Adjusted p-value
MDIST	0.061 ± 0.022	4.201 ± 1.471	0.681	< 0.001
RDIST	0.261 ± 0.094	15.620 ± 5.373	0.632	< 0.001
RMS-DIST	0.072 ± 0.027	4.869 ± 1.661	0.686	< 0.001
MV	0.337 ± 0.117	9.464 ± 3.390	0.712	< 0.001
SA	0.007 ± 0.004	11.630 ± 8.140	0.719	< 0.001
CEA	0.029 ± 0.018	174.938 ± 143.387	0.731	< 0.001
ε	0.929 ± 0.049	0.876 ± 0.095	0.582	< 0.001
MF	0.947 ± 0.312	0.383 ± 0.149	0.449	< 0.001
CF	2.981 ± 1.030	2.123 ± 0.817	0.175	0.147
DF	0.996 ± 0.002	0.997 ± 0.002	0.417	< 0.001

Abbreviations: CEA = ellipse area at the 95% confidence level; CF = centroidal frequency; DF = dispersion frequency; MDIST = mean distance; MF = mean frequency; MV = mean velocity; r = Spearman correlation coefficient; RDIST = range of distance; RMS-DIST = root mean square of distance; SA = sway area; SD = standard deviation; ε = ellipse eccentricity.

Table S5. Construct validity of postural sway parameters derived using the IMU sensor during eyes-open and eyes-closed conditions, reporting mean ± standard deviation (SD) values together with results of the Wilcoxon signed-rank test (adjusted p-value) and rank-biserial correlation coefficients (r_β).

Postural sway parameters	Eyes-open Mean ± SD	Eyes-closed Mean ± SD	Adjusted p-value	r_β
MDIST	0.052 ± 0.021	0.061 ± 0.022	0.003	-0.412
RDIST	0.217 ± 0.077	0.261 ± 0.094	0.003	-0.451
RMS-DIST	0.061 ± 0.023	0.072 ± 0.027	0.003	-0.427
MV	0.303 ± 0.099	0.337 ± 0.117	< 0.001	-0.604
SA	0.006 ± 0.003	0.007 ± 0.004	0.003	-0.481
CEA	0.021 ± 0.013	0.029 ± 0.018	< 0.001	-0.540
ε	0.900 ± 0.098	0.929 ± 0.049	0.120	-0.222
MF	1.016 ± 0.412	0.947 ± 0.312	0.109	0.214
CF	3.364 ± 1.323	2.981 ± 1.030	0.086	0.260
DF	0.997 ± 0.001	0.996 ± 0.002	0.776	0.036

Abbreviations: CEA = ellipse area at the 95% confidence level; CF = centroidal frequency; DF = dispersion frequency; MDIST = mean distance; MF = mean frequency; MV = mean velocity; r_β = rank-biserial correlation; RDIST = range of distance; RMS-DIST = root mean square of distance; SA = sway area; SD = standard deviation; ε = ellipse eccentricity.

Table S6. Construct validity of postural sway parameters derived using the force platform during eyes-open and eyes-closed conditions, reporting mean ± standard deviation (SD) values together with results of the Wilcoxon signed-rank test (adjusted p-value) and rank-biserial correlation coefficients (r_β).

Postural sway parameters	Eyes-open Mean ± SD	Eyes-closed Mean ± SD	Adjusted p-value	r_β
MDIST	3.357 ± 1.132	4.201 ± 1.471	< 0.001	-0.620
RDIST	12.146 ± 3.569	15.620 ± 5.373	< 0.001	-0.729
RMS-DIST	3.848 ± 1.270	4.869 ± 1.661	< 0.001	-0.670
MV	6.654 ± 1.718	9.464 ± 3.390	< 0.001	-0.941
SA	6.762 ± 3.542	11.630 ± 8.140	< 0.001	-0.828
CEA	108.075 ± 71.935	174.938 ± 143.387	< 0.001	-0.622
ε	0.868 ± 0.103	0.876 ± 0.095	0.714	-0.060
MF	0.343 ± 0.125	0.383 ± 0.149	0.014	-0.338
CF	2.072 ± 0.774	2.123 ± 0.817	0.714	-0.057
DF	0.997 ± 0.003	0.997 ± 0.002	0.055	0.277

Abbreviations: CEA = ellipse area at the 95% confidence level; CF = centroidal frequency; DF = dispersion frequency; MDIST = mean distance; MF = mean frequency; MV = mean velocity; r_β = rank-biserial correlation; RDIST = range of distance; RMS-DIST = root mean square of distance; SA = sway area; SD = standard deviation; ε = ellipse eccentricity.
